# Modifying an immunogenic epitope on a therapeutic protein: a step towards an improved system for antibody-directed enzyme prodrug therapy (ADEPT)

**DOI:** 10.1038/sj.bjc.6601888

**Published:** 2004-05-25

**Authors:** A Mayer, S K Sharma, B Tolner, N P Minton, D Purdy, P Amlot, G Tharakan, R H J Begent, K A Chester

**Affiliations:** 1 Cancer Research UK Targeting and Imaging Group, Department of Oncology, Royal Free and University College Medical School, University College London, Rowland Hill Street, London NW3 2PF, UK; 2 Department of Immunology, Royal Free and University College Medical School, University College London, Rowland Hill Street, London NW3 2PF, UK; 3 Institute of Infections, Immunity and Inflammation, University of Nottingham, Floor C, West Block, Queens Medical Centre, Nottingham NG7 2UH, UK; 4 Health Protection Agency, Porton Down, Salisbury, Wiltshire SP4 0JG, UK

**Keywords:** ADEPT, immunogenicity, carboxypeptidase G2, antibody response

## Abstract

Carboxypeptidase G2 (CP) is a bacterial enzyme, which is targeted to tumours by an antitumour antibody for local prodrug activation in antibody-directed enzyme prodrug therapy (ADEPT). Repeated cycles of ADEPT are desirable but are hampered by human antibody response to CP (HACA). To address this, we aimed to identify and modify clinically important immunogenic sites on MFECP, a recombinant fusion protein of CP with MFE-23, a single chain Fv (scFv) antibody. A discontinuous conformational epitope at the C-terminus of the CP previously identified by the CM79 scFv antibody (CM79-identified epitope) was chosen for study. Modification of MFECP was achieved by mutations of the CM79-identified epitope or by addition of a hexahistidine tag (His-tag) to the C-terminus of MFECP, which forms part of the epitope. Murine immunisation experiments with modified MFECP showed no significant antibody response to the CM79-identified epitope compared to A5CP, an unmodified version of CP chemically conjugated to an F(ab)_2_ antibody. Success of modification was also demonstrated in humans because patients treated with His-tagged MFECP had a significantly reduced antibody response to the CM79-identified epitope, compared to patients given A5CP. Moreover, the polyclonal antibody response to CP was delayed in both mice and patients given modified MFECP. This increases the prospect of repeated treatment with ADEPT for effective cancer treatment.

Antibody-directed enzyme prodrug therapy (ADEPT) is an experimental cancer treatment in which an enzyme linked either chemically or genetically to tumour-targeting antibody is given intravenously. When the antibody–enzyme has cleared from the circulation, a prodrug is given and is converted to active drug in the tumour by the targeted enzyme (Bagshawe *et al*, 1988). An illustration of the ADEPT concept is shown in Figure 1

**Figure 1:**
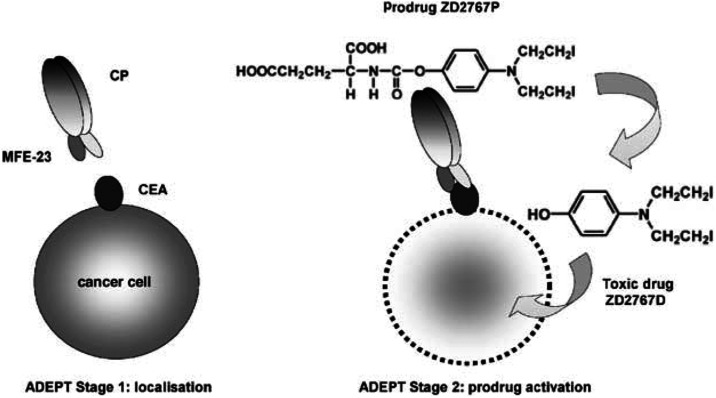
In the first stage of ADEPT, the intravenously administered antibody–enzyme fusion protein MFECPHis is allowed to localise in the tumour. In the second stage, prodrug ZD2767P, based on a bis-iodo-phenol mustard is given after clearance of enzyme from the circulation. CP cleaves the glutamate moiety to generate the active drug ZD2767D.

. ADEPT aims to overcome the shortcomings of systemic cancer treatment such as lack of tumour selectivity and drug resistance. Clinical ADEPT studies (Bagshawe *et al*, 1995; Napier *et al*, 2000; Francis *et al*, 2002) have used the bacterial enzyme carboxypeptidase G2 (CP) conjugated to A5B7, a monoclonal anticarcinoembryonic antigen (CEA) F(ab)_2_. This antibody–enzyme conjugate, termed A5CP, has demonstrated feasibility of ADEPT and evidence of therapeutic response in patients with advanced CEA-expressing adenocarcinomas. The use of CP has advantage over human enzymes in that there is no human enzyme with equivalent substrate specificity so that the danger of prodrug activation by host enzyme is avoided. However, the development of human anti-CP antibody (HACA) and human anti-mouse antibody (HAMA) was a serious limitation of ADEPT with A5CP. HACA were observed in 97% and HAMA in 100% after single administration of A5CP (Napier *et al*, 2000; Francis *et al*, 2002). Hence, methods of reducing immunogenicity of the antibody–enzyme molecule are required since neutralising antibodies can inactivate therapeutics (Baert *et al*, 2003) and are potentially detrimental to the patient. Furthermore, protein therapeutics are of increasing importance in the treatment of cancer, so a means to reduce antibody response is likely to be applicable to other therapeutic proteins.

This manuscript explores modification of immunogenic B-cell epitopes as a way to address immunogenicity of protein therapeutics. It was postulated that identification and silencing of epitopes recognised by the human immune system could allow repeated treatment with ADEPT if these epitopes could be disrupted without compromise of enzyme activity. Previously, we developed a system for identifying B-cell epitopes and applied it to identify a discontinuous conformational epitope on CP with the CM79 scFv antibody (CM79-identified epitope; Spencer *et al*, 2002) (Figure 2

**Figure 2:**
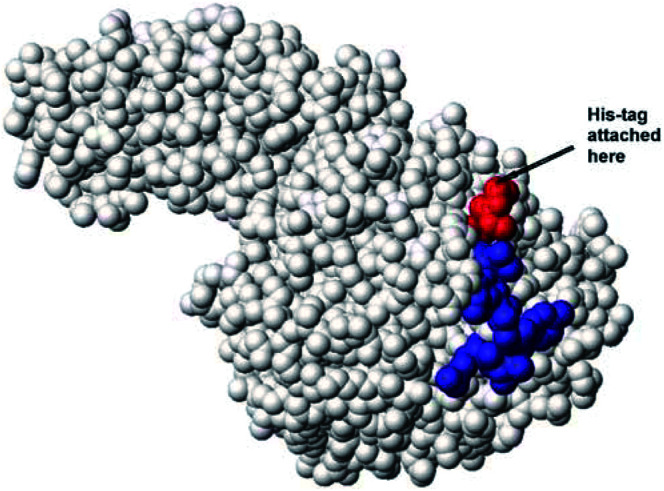
Model of CP showing the discontinuous CM79-identified epitope (region 1 residues 157–163: KEYGVRD coloured in blue, region 2 residues 412–415 coloured in red). The graphic representation was produced with MOLMOL (Koradi *et al*, 1996).

). In the current paper we modify the CM79-identified epitope to test our hypothesis.

The antibody–enzyme molecule chosen for our studies is MFECP, a fusion protein of CP with the single–chain Fv anti-CEA antibody, MFE-23 (Chester *et al*, 1994; Mayer *et al*, 2000). MFECP contains the same CP as A5CP but has advantage over A5CP in that MFECP is a recombinant molecule and therefore readily manipulated to disrupt the CM79-identified epitope. Modification of the CM79-identified epitope on the CP moiety of MFECP is proposed as a means of producing a recombinant antibody–enzyme fusion protein which retains activity but lacks the B-cell epitope. This was addressed firstly, by inserting mutations into region 1 (amino acids 157–163) and region 2 (amino acids 412–415) of the CM79-identified epitope and secondly by adding a His-tag to the C-terminus, which comprises part of region 2 (Figure 2). The success of these modifications has been tested by measuring retention of enzyme activity, testing reactivity with immune murine and human sera, investigating potential for repeated therapy in mice and, with one of the constructs, in a clinical trial.

## MATERIALS AND METHODS

### Construction, expression and purification of proteins in *Escherichia coli*

**Figure 3:**
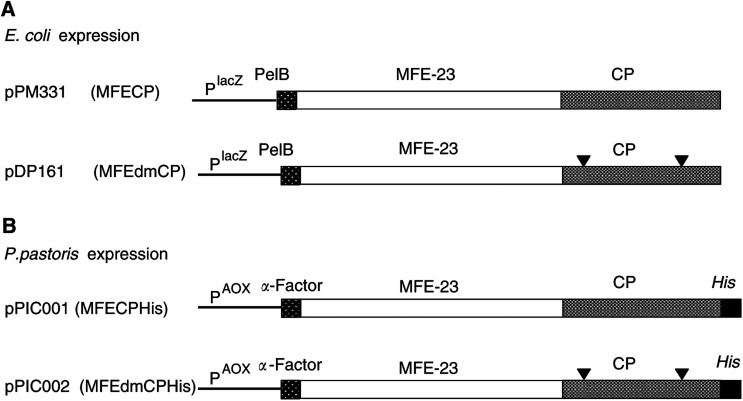
Schematic representation of the variants of MFE-CP. Panel **A** illustrates the genetic constructs of the fusion proteins expressed in *E. coli* after transfection with pPM331 (MFECP) and pDP161 (MFEdmCP), panel **B** the fusion proteins expressed in *P. pastoris* with a C-terminal His-tag after transfection with pPIC001 (MFECPHis) and pPIC002 (MFEdmCPHis).

Details of plasmid constructs are shown in Figure 3

. Construction of pPM331 (encoding MFECP, a recombinant fusion protein of the anti-CEA scFv MFE-23 and CP, an enzyme derived from *Variovorax paradoxus*, formerly *Pseudomonas* sp RS 16) has been described previously (Michael *et al*, 1996). Plasmid pDP161 (encoding MFEdmCP) is identical to MFECP except for a mutation in both regions encoding the discontinuous immunodominant epitope of CP (R162A and G412A) (Spencer *et al*, 2002). This plasmid was created by isolating a 244 bp *Sal*I/*Eco*RI fragment from the previously constructed vector carrying G412A (pDP132), and inserting it between the equivalent sites of plasmid pDP130, which carries R162A. This *Sal*I site corresponds to that found at position 1204 of the CP gene (Accession No. M12599), whereas the *Eco*RI site was created immediately following the TGA stop codon. In relation to Accession No. M12599, the nucleotide substitutions resulting in alanine replacement equate to substitution of CGC (nt 678–680) with GCG (R162A), and substitution of the G at nt 1429 with C (G412A). The pPM331 and pDP161 plasmids were expressed in *E. coli* TG1 cells and MFECP and MFEdmCP protein were purified on activated CH Sepharose 4B crosslinked to CEA as described previously (Bhatia *et al*, 2000). The eluted samples were pooled and dialysed overnight against four changes of PBS and 20 × concentrated at 4°C in a stirred cell ultrafiltration system (Amicon, UK) using a YM10 membrane (Millipore, UK). Further purification was carried out by size exclusion chromatography using a 120 ml Superdex 200 column on an automated ÄKTA purification system (Amersham Pharmacia Biotech, UK). Samples were concentrated to approximately 0.15 mg ml^−1^ and stored at −80°C. Purity and identity were confirmed by sodium dodecylsulphate–polyacrylamide gel electrophoresis (SDS – PAGE) and Western blot, CEA binding was confirmed by enzyme-linked immunosorbent assay (ELISA) (Bhatia *et al*, 2000).

### Construction, expression and purification of proteins in *Pichia pastoris*

MFECP was subcloned into the *E. coli*/*P. pastoris* shuttle vector pPICZ*α*B to enable expression in *P. pastoris*. A His-tag was engineered at the C-terminus of MFECP to facilitate purification using immobilised metal affinity chromatography (IMAC). The resultant plasmid construct (pPIC001) is shown in Figure 3. Plasmid pPIC002 is identical to pPIC001 except that it harbours the sequence encoding MFEdmCP instead of MFECP. A His-tag-encoding sequence was added to the MFEdmCP-encoding sequence on pDP161 via PCR. Two primers (Sigma Genosys, UK) were used: a 5′ primer (31 bp) that was designed to amplify the sequence from position 1696, starting 18 bp upstream of the *Sal*I site (MFECP-sense, 5′GAA GGC GGC AAG AAG CTG GTC GAC AAG GCG G) and a 3′ primer (CP-dmXbaHis anti-sense, 3′ACC TGT AAC TGC AGA ATT **C****TA GAT** TAT TAA TGG TGA TGA TGG TGA TGC TTG CCG GCG GCC AGA TCC ATG) which was complementary from position 1929 to 1947. Additional sequence was added to encode a His-tag, *Eco*RI (underlined) and *Xba*I (bold) sites and an additional tail to enhance cloning. *Eco*RI*/Sal*I digestion of pDP161 was performed prior to amplification by polymerase chain reaction (PCR). Reaction mixture comprised: 10 pmol MFECP-sense and 10 pmol CP-dm-XbaHis anti-sense primer, 128 *μ*M dNTPs (Applied Biosystems, UK), 2.5 U *Taq* Polymerase (Applied Biosystems, UK) and 10 *μ*l 10 × reaction buffer containing 15 mM MgCl_2_ (Applied Biosystems, UK) in a final reaction volume of 100 *μ*l. Five minutes at 95^o^C were followed by 30 PCR cycles consisting of 1 min at 94°C, 1 min at 75°C, 1 min at 72°C and a final extension time of 7 min at 72^o^C. The PCR product was purified with Wizard PCR preps DNA purification kit (Promega, UK) according to the manufacturer's instructions and ligated into pCR2.1TOPO (Invitrogen, UK) for nucleotide sequence confirmation (MWG Biotech, Germany). The new plasmid was digested with *Eco*RI/*Sal*I and the excised fragment was religated into the complementary sites of pDP161 yielding pDP161His. Finally, pDP161His was digested with *Xba*I and *Sfi*I and the fragment encoding MFEdmCPHis was ligated into the complementary sites of pPICZ*α*B yielding pPIC002 (encoding MFEdmCPHis).

The constructs pPIC001 (MFECPHis) and pPIC002 (MFEdmCPHis) were linearised with *Pme*I, electroporated into *P*. *pastoris* X33 on a gene pulser (Biorad, UK) and plated on YPDS agar (1% yeast extract, 2% peptone (Difco, UK) 2% glucose (Sigma, UK), 1 M sorbitol (Sigma, UK), 2% agar (Difco, UK)) containing 100 *μ*g ml^−1^ Zeocin (Invitrogen, UK). The correct insert was confirmed by PCR screening and nucleotide sequence confirmation (MWG Biotech, Germany). Cells were treated with lyticase (5 U *μ*l^−1^; Sigma, UK) to break the yeast cell wall prior to the PCR with 25 pmol 5′AOX1 primer and 25 pmol 3′AOX1 primer (Invitrogen, UK), 5 *μ*l 10 × reaction buffer containing 15 mM MgCL_2_, 64 *μ*M dNTP, 5 U *Taq* polymerase and 5 *μ*l cell lysate in a final volume of 50 *μ*l. Five minutes at 95°C were followed by 30 PCR cycles of 1 min at 95°C, 1 min at 54°C and 1 min at 72°C with a final extension time of 7 min at 72°C. A seed lot was prepared, one colony was grown in shake flasks with 20 ml YPD at 30°C (1% yeast extract, 2% peptone, 2% glucose) until an optical density of 25 at 600 nm was reached, cells were spun for 10 min at 4000 r.p.m., resuspended in YPD containing 15% glycerol and frozen at −80°C.

Large scale production of *P. pastoris* X33::pPIC001 or *P. pastoris* X33::pPIC002 expressing MFECPHis or MFEdmCPHis, respectively, were performed. A 2-l shake flask containing 200 ml YEPD/glucose medium (1% yeast extract, 2% peptone and 1.5% glucose) was inoculated with 1 ml of seed lot and incubated overnight at 30°C in an orbital shaker at 250 r.p.m. Subsequently, 5 ml of this culture was used to inoculate a second 2-l shake flask containing 330 ml of minimal fermentation medium (0.857 g CaSO_4_ (BDH, UK), 13.90 g K_2_SO_4_ (Sigma, UK), 11.14 g MgSO_4_·7 H_2_O (Sigma, UK), 8.57 g (NH_4_)_2_SO_4_ (BDH, UK), 47.6 ml glycerol (BDH, UK), 23.8 g NaPO_3_ (BDH, UK) and 3.8 ml trace element solution (Amresco, UK)). Incubation was continued as before. This culture was used to inoculate a fermentor (Bioflow 3000, New Brunswick, UK). Fermentation was performed at 30°C, pH 5 and regulated by titration with 100% NH_4_OH (Sigma, UK), 10% H_3_PO_4_ (BDH, UK) and 40% dissolved oxygen. After depletion of glycerol (carbon source) the pH was shifted to 6.5, and a limited glycerol feed was initiated, which was subsequently replaced by a limited methanol feed of 45 ml h^−1^ to induce expression of fusion proteins via the AOX promoter. Cells were harvested after 72 h by centrifugation at 4000 r.p.m. and 1 l of supernatant was purified by IMAC (Casey *et al*, 1995) on a 30 ml chelating sepharose fast flow column loaded with 0.1 M CuSO_4_. Endotoxins were removed on a 10 ml polymyxin column (Pierce, UK) according to the manufacturer's instructions. SDS – PAGE, Western blot and ELISA were performed to confirm expression of the correct protein and binding to CEA (Bhatia *et al*, 2000).

### Catalytic activity of CP

Carboxypeptidase G2 cleaves methotrexate (MTX) to give measurable changes in absorbance, which are related to enzyme concentration. This change in absorbance of MTX after CP hydrolysis was measured by spectrophotometry at 320 nm (Beckman DU-64 spectrophotometer, Soft Pac Module KINETICS Software Package, Beckman Instruments Ltd, Bucks, UK) as previously described (Minton *et al*, 1984). Measurements were performed in duplicate, 10 *μ*l of sample were incubated in 1 ml of assay buffer (100 mM Tris-HCl, pH 7.3, 0.2 mM ZnSO4 and 60 mM MTX) at 37°C. Enzyme activity was expressed in units (U), where 1 U is the amount of enzyme required to hydrolyse 1 mmol of MTX per minute at 37°C. Carboxypeptidase G2 (1000 U ml^−1^) was used as reference standard.

### CM79 scFv antibody

The anti-CP scFv antibody CM79 was expressed in *E. coli* TG1 cells with a C-terminal His-tag for purification on IMAC as described previously (Spencer *et al*, 2002). The cell-free supernatant was concentrated and purified on IMAC (Casey *et al*, 1995) on an automated ÄKTA purification system with a final yield of purified CM79 of approximately 12 mg l^−1^ culture supernatant. Purity and identity were confirmed by SDS–PAGE and Western blot. A band at the expected molecular weight of 27 kDa consistent with CM79 was detected (data not shown). The purified CM79 antibody was used in a competition ELISA to measure the proportion on immunised mice antibodies that were directed against the CM79-identified epitope (ELISA E). For later experiments, CM79 was biotinylated using the ECL biotinylation kit (Amersham, UK) according to the manufacturer's instruction. In order to check that CM79 antibody binding was not compromised by biotinylation, the binding of biotinylated and nonbiotinylated CM79 was compared using ELISA (A). Biotinylated CM79 antibody was also used to test disruption of the CM79-identified epitope in ELISA (B) and in competition assays to measure the proportion of human antibodies that were directed against the CM79-identified epitope in ELISA (C).

### Murine immunisation studies

Two separate experiments were performed, the first comparing MFECPHis (endotoxin <0.1 EU ml^−1^). and MFEdmCPHis (endotoxin 1.1 EU ml^−1^) and the second experiment comparing MFECPHis and A5CP (endotoxin <0.1 EU ml^−1^). A5CP was supplied by Lonza Biologics (UK) for the previous clinical trial (Francis *et al*, 2002) and consists of an F(ab′)_2_ derived from the affinity-purified monoclonal anti-CEA antibody A5B7 covalently linked to CP (Melton *et al*, 1993). Groups of eight Balb/c mice were injected 4 × intraperitoneally (i.p.) with 50 *μ*g A5CP, MFECPHis or MFEdmCPHis every 14 days. Mice were bled prior to each injection and 14 days after the final injection. All experiments were performed with ethical committee approval and met the standards of the UK Coordinating Committee on Cancer Research Guidelines for the welfare of animals (Workman *et al*, 1998). The resulting murine antibody response to CP was assessed using ELISA (D). Murine antibody formation to the CM79-identified epitope was assessed using ELISA (E).

### Human immune response

Sera were obtained between 21 and 43 days after treatment with 3000 U m^−2^ A5CP (Francis *et al*, 2002) or 5000 U m^−2^ MFECPHis followed by the prodrug ZD2767P for ADEPT. Both studies were approved by the local research ethics committee and informed consent was obtained from all patients. HACA formation in patients was measured by ELISA (F). Three standard deviations were added to the mean of a pool of pretreatment sera diluted 1/100 to give the cutoff for HACA-positive sera. The proportion of HACA that was directed to the CM79-identified epitope was measured by competion ELISA (G).

### ELISAs

The CP-binding capacity of human, murine and scFv antibodies was measured by ELISA. Samples in all ELISAs were applied in duplicate. Blocking to prevent unspecific binding was performed overnight with 5% milk powder in 0.1% Tween 20 in PBS containing 0.02% NaN_2_ at 4°C. Incubation times of sera and antibodies were 1 h at room temperature and wash steps consisted of 2 × PBS/0.1% Tween 20 followed by 4 × H_2_O. Peroxidase (POX) was detected with *o*-phenyldiamine (Sigma, UK) in phosphate-citrate buffer with sodium perborate (pH 5; Sigma, UK), the reactions were quenched after 5 min reaction time with 4 M HCl and plates were read at 490 nm. Alkaline phosphatase (AP) was detected in all ELISAs with *p-*nitrophenylphosphate (Sigma, UK) in 0.1 M glycine buffer (1 mM MgCl_2_, 1 mM ZnCl_2_; pH 10.4) and plates were read at 405 nm on an automated plate reader (Thermo Life Sciences, UK) after 15 min reaction time. Individual ELISA methods varied in order to measure the following:

#### ELISA (A): CP binding of biotinylated CM79 antibody

Binding of biotinylated and nonbiotinylated CM79 antibody was compared by ELISA on CP-coated plates (10 *μ*g ml^−1^ in carbonate bicarbonate buffer pH 9.2; Sigma, UK). Ten-fold serial dilutions of biotinylated and nonbiotinylated CM79 (6.5 *μ*g ml^−1^) were added, followed by anti-His antibody (1/500; Qiagen, UK) and POX-labelled sheep anti-mouse antibody (1/1000; Sigma, UK).

#### ELISA (B): Disruption of CM79-identified epitope

Reactivity of fusion proteins with CM79 was tested on plates coated with CEA (1 *μ*g ml^−1^ in PBS). Fusion proteins (150 ng well^−1^) were applied prior to biotinylated CM79 (1.3, 0.65 and 0.13 *μ*g ml^−1^). This was followed by incubation with Extravidin AP (1/5000; Sigma, UK).

#### ELISA (C): Clinical relevance of CM79-identified epitope

Clinical relevance of CM79-identified epitope was tested by comparing the immunoreactivity of HACA positive patients’ sera with MFECP and MFEdmCP on plates coated with CEA (1 *μ*g ml^−1^ in PBS) followed by MFECP or MFEdmCP (0.75 *μ*g ml^−1^). This was followed by incubation of 10-fold serial dilutions of HACA positive sera after treatment with A5CP and by AP-labelled anti-human *γ*-chain F(ab)_2_ antibody (1/10 000; Sigma, UK). Reactivity of individual sera with MFECP and MFEdmCP was tested on the same plate. Plates were read after 30 min reaction time.

#### ELISA (D): Murine polyclonal anti-CP response

Mouse antibodies to CP were measured on plates coated with CP (10 *μ*g ml^−1^ in carbonate bicarbonate buffer pH 9.2; Sigma, UK). Incubation with mouse sera (diluted 1/100) was followed by AP-labelled anti-mouse Fc antibody (1/2500; Sigma, UK). Ten-fold serial dilutions of the monoclonal anti-CP antibody SB43 (3 *μ*g ml^−1^) was used as positive control. Plates were read after 30 min reaction time.

#### ELISA (E) CM79 antibody competition with immunised mouse sera

The ability of immunised mouse sera to compete with CM79 antibody was tested on CP-coated plates in a competitive binding assay. Comparison of binding of polyclonal anti-CP antibodies in the presence and absence of CM79 antibody was measured. Mouse sera (diluted 1/100) were applied with and without CM79 antibody (25 *μ*g ml^−1^), AP labelled anti-mouse Fc antibody (1/2500; Sigma, UK) was used for detection of binding of polyclonal anti-CP antibodies in the presence and absence of CM79 antibody.

#### ELISA (F): HACA formation in patients

The human polyclonal response to CP after treatment with A5CP was measured by ELISA on CP-coated plates as described previously (Sharma *et al*, 1992). Briefly, HACA formation in patients after MFECPHis was measured on CP coated plates (10 *μ*g ml^−1^ in carbonate bicarbonate buffer pH 9.2; Sigma, UK). Incubation of 10-fold serial dilutions of sera was followed by POX-labelled anti-human *γ*-chain F(ab)_2_ antibody (1/2000; Sigma, UK).

#### ELISA (G): CM79 antibody competition with immunised human sera

ELISAs were carried out on (a) CP (10 *μ*g ml^−1^ in carbonate bicarbonate buffer pH 9.2; Sigma, UK)-coated plates and (b) CEA (1 *μ*g ml^−1^ in PBS)-coated plates followed by A5CP or MFECPHis (150 ng well^−1^) to ensure that the result was not influenced by a conformational change on CP-coated plates. Patients’ sera (1/100) were spiked with biotinylated CM79 antibody (0.65 *μ*g ml^−1^). Sera were subsequently applied, in both assays, in 10-fold serial dilutions, followed by incubation with Extravidin AP (1/5000). Inhibition of CM79 antibody binding was taken as evidence that patients’ sera had antibodies directed against the CM79-identified epitope. Binding of biotinylated CM79 antibody in spiked patients’ sera was compared to binding of biotinylated CM79 antibody in the presence of HACA-negative serum.

### Statistics

A paired Student's *t*-test was used to compare binding of post-treatment sera to MFECP and MFEdmCP and binding of CM79 to fusion proteins. The anti-CP antibody response in the murine immunisation studies and HACA formation in patients after administration of A5CP and MFECPHis was compared by Fisher's exact test. Wilcoxon signed ranks test was used to compare the murine antibody response to the CM79-identified epitope. An unpaired Student's *t*-test assuming equal variances was used for the comparison of the immune response to the CM79-identified epitope after treatment with A5CP and MFECPHis.

## RESULTS

### Modification of the CM79-identified epitope by mutation

**Figure 4:**
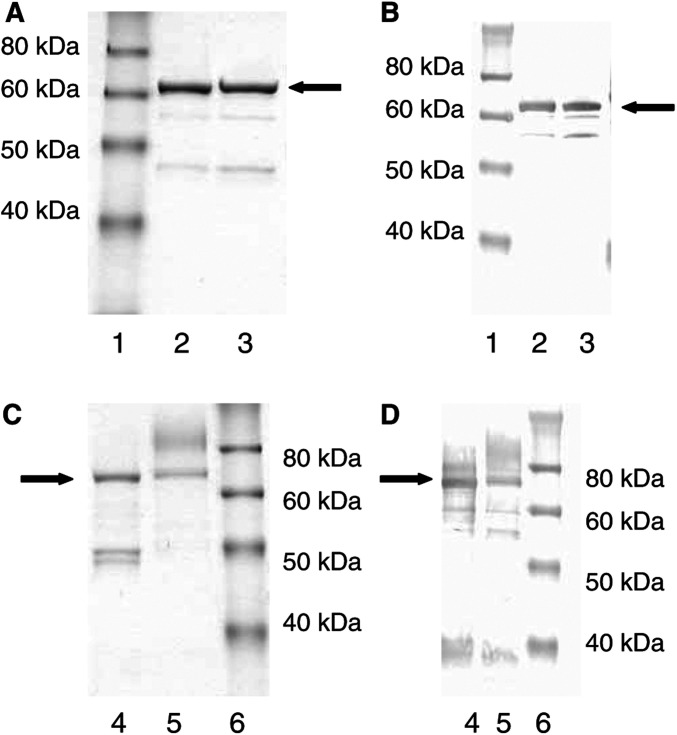
(**A**) SDS – PAGE and (**B**) Western blot of fusion proteins expressed in *E. coli* (1) molecular weight markers (2) MFECP (3) MFEdmCP. (**C**) SDS – PAGE and (**D**) Western blot fusion proteins expressed in *P. pastoris* (4) MFECPHis (5) MFEdmCPHis (6) molecular weight markers. Major band representing fusion proteins indicated by arrow.

The immunogenic CM79-identified B-cell epitope of CP was modified by mutating both of its discontinuous regions on the MFECP fusion protein. Region 1 of the CM79-identified epitope that spans amino acids 157–163 was modified by changing arginine 162 to alanine (R162A), Region 2, which spans amino acids 412–415 at the C-terminus of the molecule was modified by changing glycine 412 to alanine (G412A). The resulting double mutant of the MFECP fusion protein was termed ‘MFEdmCP’. MFEdmCP was expressed in *E. coli* and purified by affinity chromatography on CEA followed by size exclusion chromatography. MFECP, which contains wild-type CP but is identical to MFEdmCP in all other aspects, was expressed and purified in parallel experiments. Analysis by SDS–PAGE and Western blot confirmed expression of MFECP and MFEdmCP as illustrated in Figure 4

where a major band is shown at 68.5 kDa, the expected molecular weight of the fusion protein. The final yield after purification was low, as only 0.13 mg l^−1^ of supernatant were recovered for MFECP and 0.1 mg l^−1^ for MFEdmCP. However, there was no loss of enzymatic activity despite the two mutations, because when the purified proteins were tested for catalytic activity 188 U mg^−1^ was measured for MFECP and 210 U mg^−1^ for MFEdmCP.

The CM79-identified epitope is defined by its binding to anti-CP scFv antibody CM79. Successful modification of the epitope is therefore measured by reduction or ablation of binding to CM79 antibody. To test whether this had occurred, 10-fold serial dilutions of the fusion proteins were reacted with CM79 antibody using ELISA on CEA coated wells. The results showed that CM79 antibody binding to MFEdmCP was reduced by 99% compared to MFECP (*P*=0.00006, paired Student's *t*-test, Table 1

**Table 1:** Binding of biotinylated CM79 to MFECP, MFEdmCP and MFECPHis

**Fusion protein**	**CM79 binding^a^** **(%)**
MFECP (no tag)	100
MFEdmCP (no tag)	0.8^b^
MFECPHis (His-tag)	54^b^
MFEdmCPHis (His-tag)	0.9^b^

a Measured by ELISA on CEA-coated plates. Binding of biotinylated CM79 was detected by Extravidin AP.

b Binding of MFEdmCP, MFECPHis and MFEdmCPHis expressed as percentage of binding of MFECP.

). These results indicate successful modification of the CM79-identified epitope by the R162A and G412A mutations because these ablated CM79 antibody binding but did not affect catalytic activity.

### Clinical relevance of the CM79-identified epitope

To test whether the modified CM79-identified epitope was recognised by the human immune system MFEdmCP was reacted with serum samples from 12 patients who had made antibodies to CP as a result of receiving ADEPT with A5CP in a previous clinical trial. Serum samples were taken 21–35 days after treatment. For these experiments, MFEdmCP or MFECP was captured on CEA-coated wells and reacted with individual patient's sera. Results showed that all these sera had lower binding to MFEdmCP than to the unmutated MFECP. The antibody binding in this patient group was reduced by a median of 15.2% (s.d.±11.2%) and was statistically significant (*P*=0.0002, paired Student's *t*-test). The results indicated that the CM79-identified epitope is immunogenic in humans because a significant portion of the human polyclonal antibody response appeared to be directed to this epitope.

### Modification of the CM79-identified epitope by addition of a His-tag

Bacterial expression of MFECP and of MFEdmCP gave sufficient material to confirm clinical relevance of the CM79-identified epitope but did not provide a practical means of obtaining large quantities of endotoxin-free fusion proteins for immunogenicity experiments in mice and for clinical trials. This was achieved by expression in the methotropic yeast *P. pastoris* under controlled conditions in a fermentor. A C-terminal His-tag was added to facilitate large-scale purification via metal affinity chromatography, bypassing the need for CEA-affinity chromatography. His-tagged, *P. pastoris* expressed, MFECP was termed MFECPHis and His-tagged, *P. pastoris* expressed, MFEdmCP was termed MFEdmCPHis. These fusion proteins were purified by IMAC. Analysis of purified proteins by SDS–PAGE and Western blot (Figure 4C and D) confirmed expression of MFECPHis and MFEdmCPHis. Both proteins were tested for enzyme activity and the presence of endotoxin. Results showed that the endotoxin content was <0.1 and 1.1 EU ml^−1^ for MFECPHis and MFEdmCPHis, respectively. The yields obtained with the *P. pastoris* system were substantially higher than those obtained with *E. coli*, 11 mg l^−1^ supernatant for MFECPHis and 12 mg l^−1^ supernatant for MFEdmCPHis, respectively. The enzyme activity was less than for bacterially expressed fusion proteins, that is, 106 U mg^−1^ MFECPhis and 42 U mg^−1^ MFEdmCPhis.

The CM79 binding ELISA was applied to test for the presence of the CM79-identified epitope on MFECPHis and MFEdmCPHis as previously applied to test the untagged *E. coli* versions of these fusion proteins. Results, shown in Table 1 demonstrated that, as for MFEdmCP, the CM79 antibody binding to MFEdmCPHis was reduced by 99% (s.d.±0.45%) compared to MFECP (*P*=0.00006, paired Student's *t*-test, Table 1). However, MFECPHis was also shown to have reduced reactivity (54%; s.d.±2.1%) with the CM79 antibody in comparison to MFECP (*P*=0.0006, paired Student's *t*-test), although the CM79 site was unmodified. This suggested a masking of the CM79 site by the His-tag and was consistent with the His-tag conferring an independent modification on the CM79 B-cell epitope.

### Murine immune response to the CM79-identified epitope

Mouse immunisation experiments were performed to test the hypothesis that the His-tag masked the CM79-identified epitope. In one experiment, the immunogenicity of His-tag modified CP was compared with that of unmodified CP in serum of mice immunised with 50 *μ*g MFECPHis or 50 *μ*g A5CP. In a separate experiment, the immunogenicity of His-tag-modified CP was compared with that of CP that had been modified by His-tag and mutation. In this case, mice were immunised with 50 *μ*g MFECPHis or 50 *μ*g MFEdmCPHis. In both experiments, immunisations were performed every 14 days and mice were bled prior to each injection and after the final injection. A CM79-competition ELISA was designed to measure if anti-CP antibodies formed in response to these immunisations were directed to the CM79-identified epitope. Results of this ELISA indicated that mice immunised with A5CP formed antibodies to the CM79-identified epitope (*P*=0.018, Wilcoxon signed ranks test, CP binding of immunised mouse serum in presence and absence of CM79 antibody), whereas mice immunised with MFECPHis or MFEdmCPHis showed no significant response to the CM79-identified epitope in the same test (*P*=1.0 and 0.24, respectively). These results indicated not only that modification of the CM79-identified epitope successfully reduced its immunogenicity in mice but that the His-tag was as effective a means of achieving this as the R162A and G412A mutations.

### Use of MFECPHis in a phase I clinical trial and the human immune response to the CM79-identified epitope

**Figure 5:**
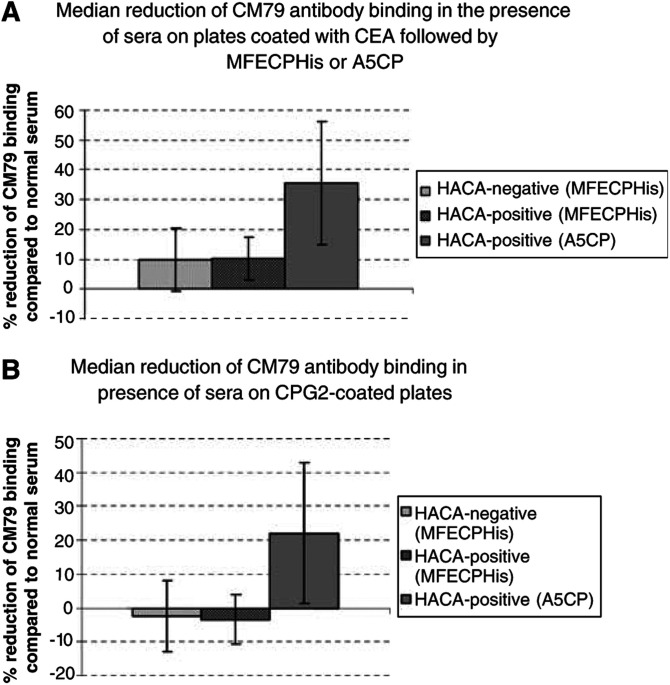
(**A**) Inhibition of binding of biotinylated CM79 antibody by sera of patients after administration of MFECPHis and A5CP. Binding of CM79 antibody in immunised sera was compared to CM79 antibody binding in normal human serum. Statistical comparison of the reduction of CM79 antibody binding after treatment with A5CP with the reduction of CM79 antibody binding after treatment with MFECPHis was significant (*P*=0.00003 for the comparison of all available sera after administration of MFECPHis and A5CP; *P*=0.023 for the comparison of HACA-positive sera only, unpaired Student's *t*-test). (**B**) Results were confirmed on CP-coated plates (*P*=0.0001 for the comparison of all available sera after administration of MFECPHis and A5CP, *P*=0.03 for the comparison of HACA-positive sera only, unpaired Student's *t*-test). Bars indicate standard deviation.

MFECPHis was chosen for the clinical trial. Murine immunisations indicated that the His-tag appeared to protect the CM79-identified epitope independently and there was no apparent advantage in further modification of the site by mutation. MFECPHis (This clinical grade MFECPHis was renamed MFECP1 to distinguish it from research grade MFECPHis.) was prepared to clinical grade, which included further purification by size exclusion chromatography and was administered to patients in a Phase I/II trial of ADEPT. Each patient received 5000 U m^−2^ of MFECPHis (approximately 50–100 mg depending on individual weight and height) followed by escalating doses of prodrug ZD2767P after clearance of MFECPHis from blood. Patients were bled at 21–42 days after treatment and their immune response to the CM79-identified epitope was analysed. Biotinylated CM79 antibody was employed for these studies, which allowed use of a simple competition ELISA testing the ability of biotinylated CM79 antibody to bind CP in the presence of patients’ sera. If the sera contain antibodies to the CM79-identified epitope, then these compete with and reduce binding of biotinylated CM79 antibody to CP on the plates. For these experiments, CP was presented to the sera/biotinylated CM79 in two different ways to minimise risk of erroneous results obtained by masking or conformational changes to epitopes when attaching to the ELISA plates. In one set of experiments, plates were coated directly with CP. In another set, plates were coated with CEA that was used to capture MFECPHis or A5CP by the antibody moiety, leaving the CP moiety of these molecules free for interaction. Three HACA-positive sera and 10 HACA-negative sera of patients treated with MFECPHis and eight HACA-positive immune sera from the A5CP ADEPT trial were available for testing in these assays, the results of which are shown in Figure 5A and B

. These results with both assays show that patients who received constructs with unmodified CP (in A5CP) made antibodies to the CM79-identified epitope through demonstration of competition of their sera with biotinylated CM79 antibody for binding to CP. However, patients who received MFECPHis did not have a response to the CM79-identified epitope as there was no significant reduction of biotinylated CM79 antibody binding to CP in the assays. The difference between the patient groups was marked (*P*=0.00003, unpaired Student's *t*-test reduced binding of biotinylated CM79 antibody in the presence of sera from patients after administration of A5CP compared to MFECPHis). This provides experimental evidence that addition of a C-terminal His-tag successfully modifies the CM79-identified epitope to reduce its immunogenicity in patients as observed with mice.

### Murine polyclonal antibody response to repeated CP administration

**Figure 6:**
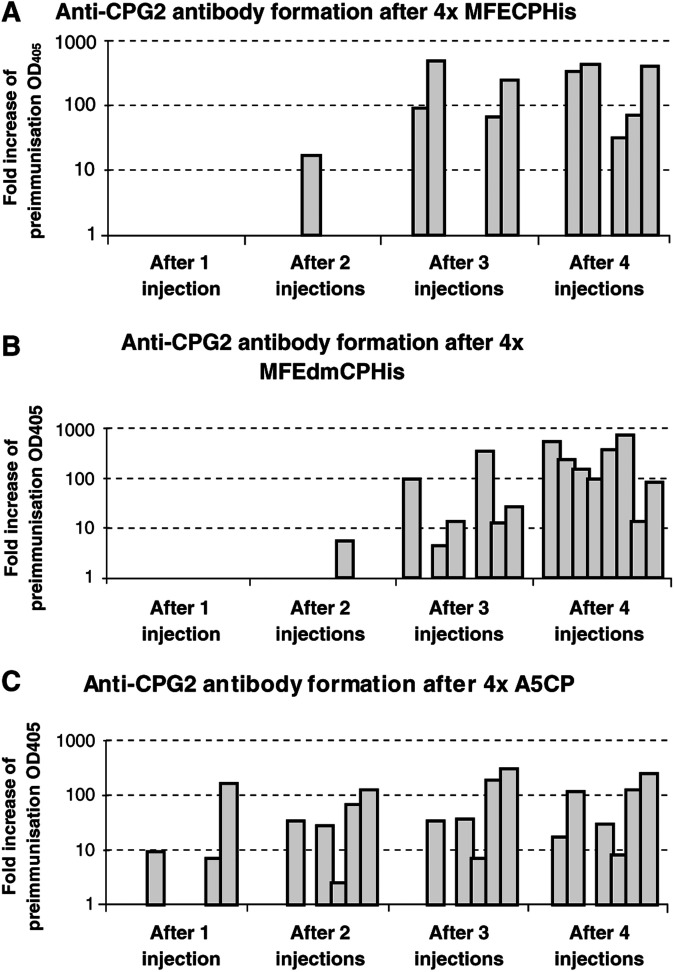
Anti-CP antibody formation in mice after 50 *μ*g MFECPHis (**A**), MFEdmCPHis (**B**) and A5CP (**C**) i.p. every 14 days for four doses. Individual bars represent mice with measurable anti-CP antibodies in each group of eight mice. Comparison of the number of injections prior to a measurable anti-CP antibody response showed no statistically significant difference between groups injected with MFECPHis and MFEdmCPHis (*P*=0.203; Fisher's exact test), while comparison of the groups immunised with A5CP and MFECPHis showed a statistical trend indicating that injections could be repeated more frequently after MFECPHis compared to A5CP (*P*=0.077; Fisher's exact test).

The polyclonal antibody response to CP was measured by ELISA 14 days after each immunising injection of MFECPHis, MFEdmCPHis or A5CP. Results from these experiments, illustrated in Figure 6

, show that Balb/c mice immunised with MFECPHis and MFEdmCPHis had no detectable anti-CP antibodies after a single injection, but developed progressively higher levels of antibody after 2, 3, and 4 immunisations with these molecules. Statistical analysis indicated that there was no significant difference in the time taken for development of mouse anti-CP antibodies to MFECPHis and the time taken for development of mouse anti-CP to MFEdmCPHis (*P*=0.203, Fisher's exact test). In contrast to these results, three out of eight mice immunised with A5CP had a detectable anti-CP antibody response after a single dose of A5CP and developed progressively higher levels after further injections (Figure 6). Statistical comparison of mice injected with MFECPHis and A5CP confirmed that injections with MFECPHis can be repeated more times than with A5CP prior to measurable antibodies to CP (*P*=0.077, Fisher's exact test).

### Human polyclonal antibody response to CP

**Figure 7:**
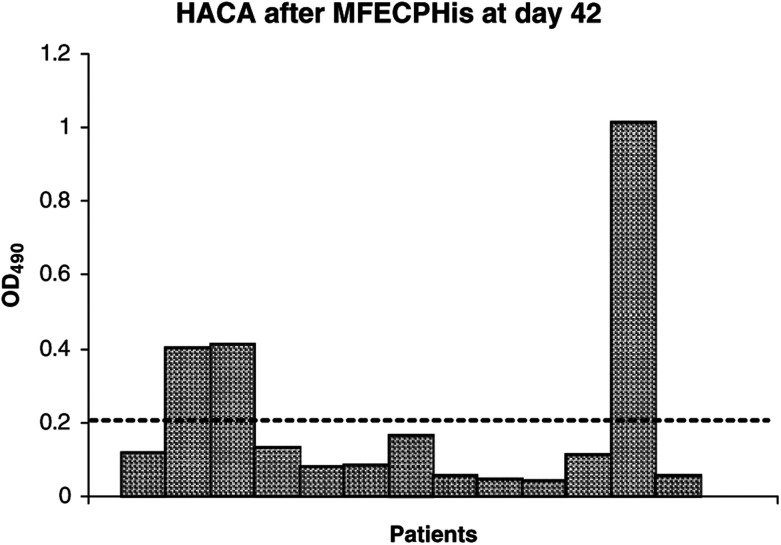
HACA formation in patients 42 days after single administration of MFECPHis, each bar represents an individual patient, the dashed line indicates the cutoff for HACA positive sera. Three standard deviations were added to the mean of a pool of pretreatment sera diluted 1/100 to give the cutoff for HACA-positive sera.

Serum of patients after treatment with MFECPHis was tested for polyclonal HACA formation using a validated anti-CP ELISA developed to monitor the ADEPT clinical trial. Results from these experiments showed that only 23% of patients (three out of 13) treated with MFECPHis had detectable HACA after a single administration of MFECPHis (Figure 7

), while 97% (36/37) of patients had detectable HACA when treated with A5CP in previous trials (Napier *et al*, 2000; Francis *et al*, 2002). Fisher's exact test confirmed statistical significance (*P*<0.0001) between the HACA response of these two patient groups.

## DISCUSSION

Protein therapeutics form an important part of cancer therapy. Antibodies, cytokines, immunotoxins and enzymes are in routine clinical use and many developing treatments are antibody-based (Carter, 2001; Glennie and van de Winkel, 2003; Hudson and Souriau, 2003). Emergence of these antibody-based therapies has been facilitated by developments in technology, which have largely addressed the limitation of immunogenicity of antibodies by methods such as chimerisation, humanisation and even production of completely human antibodies (Winter and Milstein, 1991; Carter, 2001; Glennie and van de Winkel, 2003). However, in strategies where the antibody arm is used to target bacterial enzymes or toxins, therapy is still limited by development of an antibody response to the effector arm. These targeted therapies have potential to greatly reduce the systemic toxicity of conventional chemotherapy or radiotherapy, but repeated administration is often required for effective therapy. The development of an antibody response involves different cells of the immune system, but the final common pathway involves expansion and differentiation of B-cells with antibody receptors capable of binding to epitopes on the protein therapeutic. Elimination of all immunogenic epitopes from a protein would be a laborious and possibly unachievable task, but a number of approaches have been pursued to circumvent the antibody response by modifying the administered protein to minimise its immunogenicity (Chirino *et al*, 2004). For example, site-specific pegylation has been successful for an immunotoxin used in cancer therapy (Tsutsumi *et al*, 2000) and may reduce the immunogenicity of streptavidin that has been used to improve therapeutic ratios in radioimmunotherapy (Chinol *et al*, 1998). Replacement of foreign sequences by human sequence has reduced, although not entirely eliminated antibody formation to therapeutic antibodies and cytokines (Richards *et al*, 1999; Casadevall *et al*, 2002; Welt *et al*, 2003). The ability to retain activity while reducing immunogenicity is a key element for success of targeted therapies and is more likely achieved for non-human proteins by mutations in immunogenic epitopes, since this requires minimal change in sequence and structure. In this manuscript, we explore a method to achieving this for MFECP.

MFECP is a multifunctional protein designed for ADEPT, a pretargeted cancer therapy. Reducing immunogenicity of this protein is a particular challenge as the immune response to CP, the bacterial enzyme, which forms part of MFECP, is vigorous. Hence repeated treatment with ADEPT, which is ultimately necessary to achieve effective therapy, has previously only been possible with cyclosporin immunosuppression, which carries the potential of unwanted side effects such as organ toxicity (Sharma *et al*, 1996). Attempts to overcome this by engineering human carboxypeptidase have been elegant in principal but ineffective *in vivo* (Wolfe *et al*, 1999).

We proposed that the immunogenicity of MFECP could best be reduced by modifying a B-cell epitope. B-cell epitopes are often located at a small number of sites on the surface of a protein and, as mentioned above epitope disruption potentially requires only a single amino-acid change. Furthermore, modification of B-cell epitopes is particularly attractive for proteins, where the number of repeat treatments is expected to be limited such as CP for ADEPT. While repeated administration with modified protein may eventually elicit an antibody response to a different set of epitopes, there are at least two different ways in which progressive development of this approach might allow clinically meaningful repetition of treatment in cancer patients with immunogenic proteins. First, modified proteins or totally different proteins with the same function could be used in rotation or sequence so that any antibody response made to a previously used protein would not neutralise a subsequent protein that shares no immunogenic epitopes. Second, is to reduce the ‘danger’ signal provided by protein antigens (Matzinger, 2002). In this situation, a reduction of strongly immunogenic epitopes for either B cells or T_h_ cells could lead to anergy or tolerance towards minor immunogenic epitopes in a noninflammatory situation. The experience with monoclonal antibodies supports this approach in that murine monoclonal antibodies were strongly immunogenic and led to elimination of the monoclonal antibodies within 14 days in most nonimmunosuppressed patients. Chimerisation of murine monoclonal antibodies still left 15% of the murine variable region sequences present in the chimeric monoclonal antibody, yet there was a dramatic fall in immunogenicity and antibody production. Indeed, clinical experience has failed to show any clinically significant benefit for humanisation in which the residual murine complementarity determining region sequences comprise 7% compared to chimerisation (Baert *et al*, 2003; Welt *et al*, 2003). This suggests that the constant region of monoclonal antibodies contain the most strongly immunogenic epitopes and its removal, although not removing all potentially immunogenic epitopes, reduces the ‘danger’ signal sufficiently that they are much less likely to trigger an antibody response.

Identification of B-cell epitopes by hybridoma technology has been laborious in the past, but feasibility has vastly improved due to the availability of a generic method for rapid recognition of B-cell epitopes based on a phage display library of scFv antibodies, surface enhanced laser desorption and ionisation (SELDI) affinity mass spectrometry and bioinformatics tools as described previously by our group (Spencer *et al*, 2002). The CM79 site, a surface-exposed conformational B-cell epitope comprising the C-terminus and an internal sequence of CP has been identified by this method and we have shown previously that the human antibody response to CP is substantially directed towards this CM79 site. In the current paper, we show that modification of the CM79-identified epitope of CP could be achieved by mutation or by addition of a C-terminal His-tag on the recombinant fusion protein MFECP. Both of these modifications resulted in reduced antibody formation to the CM79-identified epitope. In addition, a delayed antibody response to CP in mouse and a significant reduction of HACA formation in man was seen.

The strategy of mutating B-cell epitopes to reduce immunogenicity has also been applied with success by other workers for staphylokinase (Collen *et al*, 1996) and L-asparaginase (Moola *et al*, 1994). This is however, to our knowledge, the first reported instance of successful masking of an immunogenic epitope by addition of a His-tag. His-tags are usually added to proteins for purification purposes and are not widely reported to affect protein function, although Goel *et al* (2000), have observed that the addition of a His-tag to a scFv antibody appeared to interfere with its antigen-binding. The experimental observations made by these workers were supported by molecular modelling and are consistent with our results that indicate an ability of His-tags to adopt conformational states, which interfere with protein–protein interactions.

Other factors affecting the formation of an antibody response to CP need nevertheless to be considered. Firstly, an immunoreactive T-cell epitope may be involved in the mutated region as recently described for B-cell epitope modified staphylokinase (Warmerdam *et al*, 2002). However, while mutation may potentially affect a T_h_-cell epitope, addition of the C-terminal His-tag is unlikely to have the same effect. Other factors known to affect neutralising antibody formation include the route of administration, dosage regimen, presence of endotoxins and aggregation (Braun *et al*, 1997). Route of administration and dosage regimen were equivalent for all tested proteins and endotoxins, which are known to increase the immunogenicity of proteins are unlikely to contribute to the differences we observed because all proteins tested in this study had a similar endotoxin content. However, MFECPHis is glycosylated with mannose following expression in *P. pastoris* and A5CP is not. Glycosylation may mask immunogenic epitopes or affect protein conformation. Also, MFECPHis cleared from circulation within 24 h, while A5CP takes several days and MFECPHis was confirmed to be monomeric, while aggregation due to the process of chemical conjugation may contribute to the antibody formation after A5CP. These parameters may contribute to the immune response to the whole molecule and will be investigated in later experiments, but do not explain the lack of the antibody response to the CM79 site.

In conclusion, reduced antibody formation to the CM79-identified epitope was observed in mice and men after modification by mutation and addition of a C-terminal His-tag. This was accompanied by a reduced antibody formation to CP, which could allow repeated treatment with ADEPT. Modification of B-cell epitopes appear to be a useful strategy for the reduction of immunogenicity to foreign proteins and may have generic application, in particular for recombinant cancer therapeutics, which are not intended for protracted use.
